# Using the Technology Acceptance Model to conceptualise experiences of the usability and acceptability of a self-management app (COPD.Pal®) for Chronic Obstructive Pulmonary Disease

**DOI:** 10.1007/s12553-020-00494-7

**Published:** 2020-11-26

**Authors:** Liam Knox, Rachel Gemine, Sarah Rees, Sarah Bowen, Phil Groom, David Taylor, Ian Bond, William Rosser, Keir Lewis

**Affiliations:** 1 University of Sheffield, SITraN, 385a Glossop Road, Sheffield, S10 2HQ UK; 2 Hywel Dda University Health Board, Carmarthen, UK; 3 Swansea University, Singleton Park, Sketty, Swansea, SA2 8PP UK; 4 Bond Digital Health Ltd., The Maltings, E Tyndall St, Cardiff, CF24 5EA UK

**Keywords:** Chronic obstructive pulmonary disease, Technology acceptance model, Self-management, User-centred design, Reflective thematic analysis

## Abstract

Chronic Obstructive Pulmonary Disease (COPD) is a long-term progressive inflammatory lung disease causing chronic breathlessness and many hospital admissions. It affects up to 1.2 million people in the UK. To help people with COPD self-manage their condition we developed, in partnership with healthcare users, a digital mobile phone app called COPD.Pal®. We report the first user feedback of COPD.Pal®, applying the Technology Acceptance Model (TAM) theoretical framework.

11 participants engaged with a click dummy version of COPD.Pal® before being asked questions relating to their experiences. A deductive, semantic, reflexive thematic analysis was conducted to analyse their individual and collective experiences. The study was registered at Clinical Trials.gov (NCT04142957).

Two overarching themes resulted: Ease of Use and Perceived Usefulness. Within the former, participants discussed how they wanted flexibility and choice in how they engaged with the app; including how often they used it. Additionally, they discussed how the app layout should make it straightforward to use, whilst unanimously agreeing that COPD.Pal® provided this. Within Perceived Usefulness, participants discussed how they wanted the information they entered into the app to be useful, in addition to the app providing resources regarding COPD. Lastly, there was disagreement regarding preferences for further app development.

We found that COPD.Pal® was usable and acceptable by people with COPD and TAM provided a useful theoretical framework for both structuring discussions with users and analysing their comments.

**Electronic supplementary material:**

The online version of this article (10.1007/s12553-020-00494-7) contains supplementary material, which is available to authorized users.

## Introduction

Chronic Obstructive Pulmonary Disease (COPD) is a global problem, with 210 million sufferers and 3 million deaths annually [1]. It is predicted to become the third most common cause of death worldwide by 2030 [2]. There are 1.2 million people with known COPD in the UK, but this is likely to be an underestimate [3]. People with COPD have daily symptoms, a poorer health status, reduced exercise capacity, and impairment in lung function [4]. Recurrent exacerbations can lead to hospital admissions and deterioration in quality of life and increased mortality [4].

Despite people with the condition being extensive users of the National Health Service (NHS) [5, 6], approximately only 1% of their time is spent with healthcare professionals [7]. The rest of the time, people with COPD are increasingly encouraged to self-manage their condition; here behaviours including regular exercise, taking prescribed medication, being aware of symptoms, and attending healthcare appointments are encouraged [8]. Supporting self-management behaviours has been highlighted as crucial for the care of people with COPD [9]. Despite the positive relationship between more self-management and better health outcomes [10], these behaviours are seldom conducted daily [11] and adherence to medication is historically low [12, 13].

**Fig. 1:**
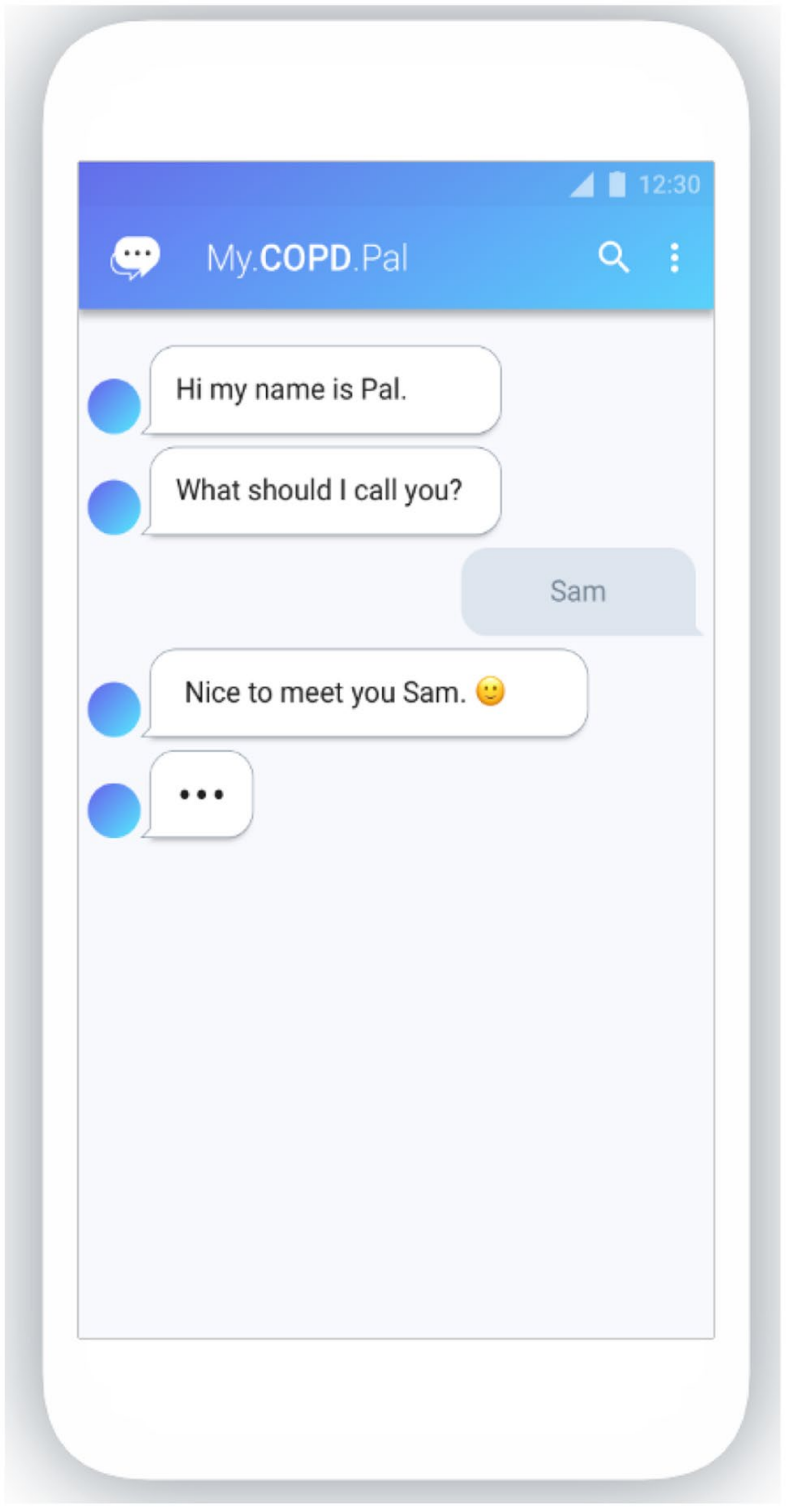
COPD.Pal's text-based system

One person with COPD created a simple diary, to enable greater awareness of the change in his symptoms and identify when he was likely to have an exacerbation and thus take preventative action. Bond Digital Health Ltd. (BDH; Cardiff, UK; https://bondhealth.co.uk/) first transformed this paper tool into an electronic diary, then a smart phone app. ‘COPD.Pal®’ aims to allow people with COPD to track and manage their condition. The interface replicates a simple text-based system (see Fig. 1), so anyone who can use SMS messaging can use it. COPD.Pal® can ask questions regarding symptoms (e.g. COPD Assessment Test [14]), wellness (e.g. quality of life, such as EQ-5D [15]), and medication usage; including increasing or decreasing use of symptom reliever inhalers. The app can also send reminders to follow individual self-management plans.

Despite healthcare mobile phone apps becoming increasingly prevalent, few of these devices have been developed through co-production with end-users or been properly evaluated in a clinical setting for people with COPD [16].

**Fig. 2:**
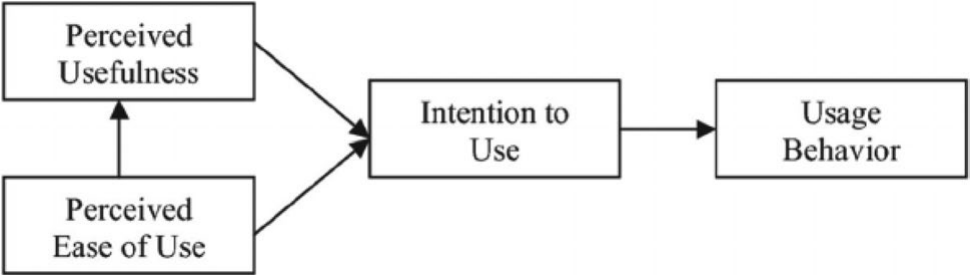
The Technology Acceptance Model (Davies, et al., 1989)

Others have attested to the value of qualitative methodology within medicine to help further understanding and knowledge [17]. Researchers and healthcare designers should apply theoretical constructs to facilitate intervention development [18] so we applied the Technology Acceptance Model (TAM; see Fig. 2) [19]. TAM posits that the greater the perceived usefulness and ease of use is, the greater behavioural intention will be and thus actual use of the technology. Additionally, perceived ease of use influences perceived usefulness, in that as the former increases, so too does the latter. These relationships within the theory are demonstrated by the arrows shown in Fig. 2. This theory has been used previously to understand acceptance of other healthcare technologies [20, 21].

This first phase study empirically explores the early development of COPD.Pal® using qualitative methodology to answer the question: *‘how do people with COPD experience the usability and acceptability of the COPD.Pal*® *app?’.*

## Methods

NHS Research Ethics Committee (19/LO/1649) and R&D approvals were received, and the study was pre-registered online (ClinicalTrials.gov - NCT04142957).

### Participants

People with COPD were identified by the clinical research team from the research COPD database (in Prince Philip Hospital, Wales), hospital clinics, pulmonary rehabilitation lists, Breathe Easy groups, and Respiratory Innovation Wales’ (https://riwales.com/) Expert Patient Network.

85 people meeting the inclusion and exclusion criteria (see Table 1) were invited to take part in the study. The team arranged a mutually beneficial date and time with 15 people, with 11 showing up on the single day. Table 2 describes the 11 who took part with the focus group.

**Table 1:** Inclusion and exclusion criteria. FEV_1_ = Forced expiratory volume during the first second, FVC = Forced vital capacity

Inclusion Criteria	Exclusion Criteria
Willing and able to provide written informed consent	Unwilling or unable to provide written informed consent
Clinical diagnosis of COPD as defined by GOLD (4): 40 years or older • At least 10 pack-years smoking history • Spirometry FEV1/FVC ratio less than 70% • FEV1 less than 80% predicted (4)	Cognitive, visual, or hearing impairment which would affect communication in a group-setting or ability to see and use a smart phone
Owns or has access to a smart phone

**Table 2:** Participant characteristics. Unless otherwise stated, all figures given show the mean value with standard deviation in parentheses. FEV_1_ = Forced expiratory volume during the first second

Characteristic	N = 11
Age (years)	66.5 (5.7)
Gender (n)	Male = 8, Female = 3
FEV_1_% predicted	52.1 (19.8)

### Procedure

Potential participants were posted an invitation letter and Participant Information Sheet (PIS) and asked to return a pre-paid reply or telephone the researchers.

The focus group was held in a location with free parking and easier access than a hospital as this was felt important by our patient and public involvement group and travel expenses were reimbursed. A written informed consent form was obtained upon arrival from each participant.

Participants engaged with the first version of the app using a click dummy (a partly interactive prototype of the application) loaded on to smart phones which were provided by BDH. The participants were given as long as necessary to use the application and were supported by three of the authors if they had any queries.

A semi-structured focus group was led by an experienced qualitative researcher with an observer and audio recorded. Questions were asked relating to the usability and acceptability of COPD.Pal® (see the Interview Schedule, Supplementary File 1). The focus group lasted approximately 60 min. Only one focus group was conducted due to time and funding restrictions.

## Analysis

Each interviewee was given a number and information that revealed their identity was removed to ensure anonymity. A verbatim account of the focus group was produced.

A reflexive thematic analysis [22, 23] was applied to allow the analysis of multiple components relating to the research area [24]. This analysis method was also most appropriate due to the sparsity of current research within technology, self-management apps for people with COPD [25]. Transcripts were coded using an inductive (data-driven) procedure, but themes were interpreted and contextualised according to concepts within the TAM (see Fig. 2). Thus, whilst the initial coding remained data-driven, focusing on the content within the transcript, the analysis adopted a more theoretically-driven approach, exploring the level to which codes fit with the theory, similar to others within a health context [26]. Semantic themes were focused upon during the analysis in an aim to identify surface-level views [27] and opinions regarding the usability and acceptability of a click-dummy version of COPD.Pal®. This is opposed to latent themes which would aim to understand underlying ideas, patterns, and assumptions [27].

## Results

We summarise how participants described their experiences of COPD.Pal®, using the TAM (see Fig. 2) overarching theoretical framework, particularly within the concepts of *Ease of use* and *Perceived usefulness*.

### Ease of use

Within this overarching theme the authors developed two sub-themes; *Flexibility and choice* and *App layout*.

Participants discussed how they wanted greater flexibility and choice with how they engaged with the COPD.Pal® app. This topic was discussed at the beginning of the focus group in addition to re-emerging organically throughout the discussion. Whilst discussing how often they wished to engage with the app, there was some disagreement within the group; however, one participant suggested being given the choice which quickly gained positive remarks from all.

“Well yes definitely I think the more choice and information you have the better, so it’s down to the individual to choose what they do and do not do”. Participant 6.

This choice was also extended to what ‘add-ons’ (i.e. extra measures and functions) should be added to the app or included in future additions. Lastly within this sub-theme, participants discussed how they did not want to lose their data if they broke their phone or bought a new one. Therefore, it was suggested that the information should be stored online so that it could be accessed by multiple devices simultaneously if necessary.

Participants also discussed how the app layout should be easy to engage with and suggested a dashboard or calendar could be beneficial to interpret data over time. The participants felt this type of layout would help both people with COPD and healthcare professionals.

Throughout the focus group, participants repeatedly stated that they would be unlikely to use the app if the data was not being used by someone. Some of participants wanted to engage with the app solely to help their healthcare professional make health-related decisions during appointments. Therefore, it was believed that if the information was presented in a too complicated fashion, the healthcare provider would then disengage which would result in the person with COPD also giving-up.

“Is the doctor or the GP going to be switched on to get that data? Or will he be trained up or whatever? Because some of the researchers would likely be able to understand it, but the local GPs probably want to go and play golf at 3 o’clock!” Participant 2.

Participants unanimously agreed that the current version of COPD.Pal® was extremely easy to use because of the layout and display of the app.

“It’s crisp, easy to read, good fonts, I suffer with severe dyslexia and I struggle to read, but that was easy to read” Participant 4.

This was seen as a large benefit as the group discussed that even those with poorer computer skills would still be able to engage with the app.

### Perceived usefulness

Within this overarching theme the authors developed three sub-themes from the transcript; *Used by anyone*, *App development*, and *Greater information*.

Participants emphasised several times that the information entered into the app had to be used by someone. Although there was disagreement regarding for whom COPD.Pal® could be most beneficial, participants agreed that providing the information was being used by someone (healthcare professional or themselves) they would continue to engage with it.

“I want to know that when I go and give [GP] the data he would use it rather than look at it and shrug” Participant 5.

“Well any information you enter [into COPD.Pal®] will be useful to someone, it’s useful to yourself. It’s not a waste of time to do it” Participant 6.

Participants did believe there were situations where COPD.Pal® could benefit both healthcare professionals and people with COPD.

“When you go and see your specialist once or twice a year, you sit down at the reception and you fill in that form every time…but now with the app you don’t have to do that and they will have access to the data…it will alleviate a lot of form filling” Participant 6.

This could reduce the time taken during clinic visits as healthcare users would not have to complete the questionnaire during the visit (as all the necessary information would already be accessible on the app) but be prompted by information already available. Several participants also highlighted that the information would be more accurate, as they would not be relying on their memory.

There was some disagreement within the sub-theme of *app development*. Several participants wanted lots of add-ons and further versions of the app to be created that would increase the perceived usefulness of the device. However, other participants maintained too many additional measures and abilities could make COPD.Pal® harder to use and decrease the likelihood of engagement.

“I think [add ons] could be very confusing and potentially make us hypochondriacs…well the more information a layman has, the more complicated and imaginary it becomes” Participant 8.

Lastly, there was discussion amongst participants regarding how they wished the app only to focus on their COPD and *not* be expanded to cover other conditions or less relevant causes of their condition.

The final sub-theme of *greater knowledge* concerned how participants wanted more information regarding the data they entered into COPD.Pal®. Participants largely agreed that such information could be included rather easily, either by having small pop-up information, or through links to a COPD.Pal® website explaining various scores.

“Well maybe if you’re not sure about whatever you could have a link to [a COPD.Pal®] website to explain in easy language what [questionnaire scores] mean. So you see ‘20′, ‘well what the hell is that?’, so you click the link and you go to the website and then it tells you what that means” Participant 6.

## Discussion

This research study examined how people with COPD experienced the usability and acceptability of COPD.Pal®, using the Technology Acceptance Model as a framework to guide the thematic analysis. We found that, in general, participants did discuss COPD.Pal® in terms of the app’s ease of use and perceived usefulness, and large sections of discourse were devoted to the interlinked relationship between these two concepts. Although TAM only states that ease of use influences perceived usefulness (as opposed to this relationship being reversed; see Fig. 2), several participants within this focus group believed that increasing the functionality of the app (i.e. increasing perceived usefulness), could negatively impact its usability. Thus, there does appear to be a balance between these two concepts, as increasing either could increase engagement with COPD.Pal®, but too much of one could be at the expense of the other component. Future research could investigate whether the relationship between TAM’s ease of use and perceived usefulness should be reconceptualised.

One element that was repeatedly emphasised throughout the focus group was the need for COPD.Pal® to be flexible and provide choice to the individual in how and how often they engaged. At several points there was disagreement between participants and the ability to choose was often considered the solution for making the app accessible for the largest number of people. The importance of providing choice to people with COPD has been identified previously by the authors (unpublished work) and has links with self-determination theory’s ‘autonomy’ [28]. Within this focus group, participants emphasised how COPD effects everyone differently, and the ability to autonomously engage with COPD.Pal® in-line with their needs and wishes was believed to be a large benefit. However, this element should not be mistaken with a desire for the app not to display reminders, as this was felt beneficial to ensure they remembered to enter data or even take medication.

Although it is hoped that people with COPD should largely be responsible for their own care through self-management [7, 9], several participants were predominately motivated to engage with COPD.Pal® to aid healthcare professionals make medical decisions for them. However, this was one element in which there was disagreement, with several participants wanting to use the data themselves. Although this study did not explore possible causes for the disagreements, one potential explanation could be how experienced the participant is with self-management techniques. It is plausible that less experienced individuals engage with the app to help others make decisions on their behalf, but more experienced individuals who have had the disease for many years or already undergone Pulmonary Rehabilitation or Expert Patient Programmes would be more comfortable using the data themselves. It is important to note, however, that one participant who identified themselves as being uninvolved with their own care, believed that entering in their data longitudinally could help them identify what exacerbated their condition and would increase their self-management and COPD knowledge. This belief quickly obtained agreement from others. This could be an important finding because it suggests that although the participants may be motivated to self-manage their condition, they do not possess the skills to actively engage with the necessary techniques. Additionally, this also represents an important aspect of COPD.Pal®, as it indicates that the original aim which prompted its development (i.e. empowering the individual), could indeed be met through the current or future versions.

Throughout the focus group, participants were extremely positive regarding COPD.Pal® and how it could be used to enable them or their healthcare professionals to make more informed decisions regarding their condition. As a result of the high acceptability of the current iteration of the app, participants indicated that they would be highly likely to continue using COPD.Pal® longitudinally (if given the opportunity) where this could see benefits for both themselves and healthcare providers.

Regarding the strengths and limitations of this study, one major strength is the involvement of people with the condition at an extremely early stage of app development. Although co-production has been promoted for its significant benefits [29] and the proclivity of medical devices being released to the public, there appears to be a sparsity of surrounding evidence regarding these technologies. This is not only detrimental for the individual, as they may be engaging with unproven or untested technological devices, but also the developers of these devices because it is likely that without addressing underlying needs, engagement may be short-lived. One weakness of this study is that we only conducted one focus group with the target population. Although we would have liked to have conducted more groups with a variety of stakeholders (e.g. healthcare providers, more people with COPD) with different characteristics (e.g. differing levels of technology experience), timing and funding pressures made this impossible. However, to overcome this limitation we recruited a relatively large sample from both primary and secondary care; thus, increasing the breadth of views we captured regarding COPD.Pal®. Additionally, neither the clinical team nor app developers were present during the focus group, which can put the participants at ease. By positioning themselves as impartial and separate to COPD.Pal®, the researchers conducting the focus group were able to elicit widespread views – both positive and negative – regarding the app and this increased the validity of our research design.

In conclusion, this study shows that a self-management app is both usable and acceptable to people with COPD and that TAM is a useful model to conceptualise how people discussed COPD.Pal®. Developers of technology healthcare interventions should be cognisant of the concepts of perceived usefulness and ease of use to increase the likelihood of engagement from the healthcare users. Failure to acknowledge TAM, or another readily accessible framework, could result in a lack of sustained engagement and the wasting of resources. This study provides a novel contribution to a field which rarely conducts or publishes rigorous early research concerning healthcare apps and highlights how significant benefits and data can be obtained for system developers through low intensity methodology. Future research regarding COPD.Pal® will utilise quantitative clinical trial methodology to further evaluate and develop the app and ensure that the system represents an effective and usable tool for people with COPD.

## Data availability statement

Unfortunately, the authors did not ask participants to provide consent for their data to be stored in an openly accessible online repository and therefore research data for this study is not shared. However, the authors invite any and all questions regarding data, which they will provide as much information as possible covered by the consent provided by participants already.

## Electronic supplementary material

Below is the link to the electronic supplementary material.

Supplementary file1 (DOCX 21.1 kb)
