# Supplementary material for: Using the Technology Acceptance Model to conceptualise experiences of the usability and acceptability of a self-management app (COPD.Pal®) for Chronic Obstructive Pulmonary Disease
Source: Health Technol (Berl). 2020 Nov 26;11(1):111–7. doi: 10.1007/s12553-020-00494-7 (PMC7690946; doi:10.1007/s12553-020-00494-7)
Supplement: Supplementary file 1 — Supplementary file1 (DOCX 21.1 kb) [file 12553_2020_494_MOESM1_ESM.docx]

## Supplementary file 1 – Interview Schedule

1. What was your experience using the app?
2. How easy was the app to use?
3. Did you have any problems with using the app?
   1. Describe/discuss
4. What would you change about the app?
   1. Anything to be added or removed?
5. Would you continue to use COPD.Pal®?
6. Do you think it could help you manage your COPD?
   1. If so, how?
7. How did you find the questions on the app?
   1. Did you understand them?
   2. Could you answer them?
8. What did you think about the number of questions?
9. What have you noticed affects your COPD?
   1. Pollen, pollution, the weather?
10. How often would you like to interact with the app?
11. What are the potential barriers and facilitators of using the app?
